# Vitamin D deficiency is associated with respiratory symptoms and airway wall thickening in smokers with and without COPD: a prospective cohort study

**DOI:** 10.1186/s12890-020-1148-4

**Published:** 2020-05-04

**Authors:** Auyon J. Ghosh, Matthew Moll, Lystra P. Hayden, Jessica Bon, Elizabeth Regan, Craig P. Hersh, the COPDGene Investigators, James D. Crapo, Edwin K. Silverman, Barry J. Make, Elizabeth A. Regan, Terri Beaty, Ferdouse Begum, Peter J. Castaldi, Michael Cho, Dawn L. DeMeo, Adel R. Boueiz, Marilyn G. Foreman, Eitan Halper-Stromberg, Lystra P. Hayden, Craig P. Hersh, Jacqueline Hetmanski, Brian D. Hobbs, John E. Hokanson, Nan Laird, Christoph Lange, Sharon M. Lutz, Merry-Lynn McDonald, Margaret M. Parker, Dandi Qiao, Elizabeth A. Regan, Edwin K. Silverman, Emily S. Wan, Sungho Won, Phuwanat Sakornsakolpat, Dmitry Prokopenko, Mustafa Al Qaisi, Harvey O. Coxson, Teresa Gray, Mei Lan K. Han, Eric A. Hoffman, Stephen Humphries, Francine L. Jacobson, Philip F. Judy, Ella A. Kazerooni, Alex Kluiber, David A. Lynch, John D. Newell, Elizabeth A. Regan, James C. Ross, Raul San Jose Estepar, Joyce Schroeder, Jered Sieren, Douglas Stinson, Berend C. Stoel, Juerg Tschirren, Edwin Van Beek, Bram van Ginneken, Eva van Rikxoort, George Washko, Carla G. Wilson, Robert Jensen, Douglas Everett, Jim Crooks, Camille Moore, Matt Strand, Carla G. Wilson, John E. Hokanson, John Hughes, Gregory Kinney, Sharon M. Lutz, Katherine Pratte, Kendra A. Young, Surya Bhatt, Jessica Bon, Mei Lan K. Han, Barry Make, Carlos Martinez, Susan Murray, Elizabeth Regan, Xavier Soler, Carla G. Wilson, Russell P. Bowler, Katerina Kechris, Farnoush Banaei-Kashani, Jeffrey L. Curtis, Carlos H. Martinez, Perry G. Pernicano, Nicola Hanania, Philip Alapat, Mustafa Atik, Venkata Bandi, Aladin Boriek, Kalpatha Guntupalli, Elizabeth Guy, Arun Nachiappan, Amit Parulekar, Dawn L. DeMeo, Craig Hersh, Francine L. Jacobson, George Washko, R. Graham Barr, John Austin, Belinda D’Souza, Gregory D. N. Pearson, Anna Rozenshtein, Byron Thomashow, Neil MacIntyre, H. Page McAdams, Lacey Washington, Charlene McEvoy, Joseph Tashjian, Robert Wise, Robert Brown, Nadia N. Hansel, Karen Horton, Allison Lambert, Nirupama Putcha, Richard Casaburi, Alessandra Adami, Matthew Budoff, Hans Fischer, Janos Porszasz, Harry Rossiter, William Stringer, Amir Sharafkhaneh, Charlie Lan, Christine Wendt, Brian Bell, Marilyn G. Foreman, Eugene Berkowitz, Gloria Westney, Russell Bowler, David A. Lynch, Richard Rosiello, David Pace, Gerard Criner, David Ciccolella, Francis Cordova, Chandra Dass, Gilbert D’Alonzo, Parag Desai, Michael Jacobs, Steven Kelsen, Victor Kim, A. James Mamary, Nathaniel Marchetti, Aditi Satti, Kartik Shenoy, Robert M. Steiner, Alex Swift, Irene Swift, Maria Elena Vega-Sanchez

**Affiliations:** 1 Channing Division of Network Medicine, Department of Medicine, Brigham and Women’s Hospital, 181 Longwood Avenue, Boston, MA 02115 USA; 2 Division of Pulmonary and Critical Care Medicine, Department of Medicine, Brigham and Women’s Hospital, Boston, MA USA; 3 Division of Respiratory Diseases, Children’s Hospital, Boston, MA USA; 4 Division of Pulmonary, Allergy, and Critical Care Medicine, Department of Medicine, University of Pittsburgh Medical Center, VA Pittsburgh Healthcare System, Pittsburgh, PA USA; 5 Division of Rheumatology, Department of Medicine, National Jewish Health, Denver, CO USA

**Keywords:** COPD, Vitamin D, Respiratory symptoms, Quantitative imaging

## Abstract

**Background:**

Previous studies have established a higher prevalence of vitamin D deficiency in patients with COPD, but the relationship between vitamin D levels and COPD exacerbations remains controversial. In addition, the effect of vitamin D levels on imaging characteristics remains mostly unexplored. Using cross-sectional and longitudinal follow up data from the COPDGene Study, we assessed the association between vitamin D levels on respiratory symptoms, exacerbations, and imaging characteristics. We hypothesized that vitamin D deficiency will be associated with worse respiratory-related outcomes.

**Methods:**

Current and former smokers between ages 45–80 were enrolled the COPDGene Study. Subjects completed questionnaires, spirometry, six-minute walk test, and chest computed tomography scans. A subset of subjects had measurement of serum concentration of 25-hydroxyvitamin D (25(OH)D). Vitamin D deficiency was defined as serum concentration less than 20 ng/mL. Longitudinal follow up was conducted via a web-based or telephone questionnaire.

**Results:**

Vitamin D levels were measured on 1544 current and former smokers, of which 981 subjects had sufficient vitamin D levels and 563 subjects had vitamin D deficiency. Subjects with vitamin D deficiency were younger with increased likelihood of being African American, being current smokers, having a lower percent predicted FEV_1_, and having COPD. Vitamin D deficiency was associated with worse quality of life, increased dyspnea, decreased exercise tolerance, and increased frequency of severe exacerbations. Vitamin D deficiency was also associated with increased segmental airway wall thickness on chest CT scans.

**Conclusion:**

Vitamin D deficiency was associated with increased respiratory symptoms, decreased functional status, increased frequency of severe exacerbations, as well as airway wall thickening on chest CT scans. Further research is needed to determine the potential impact of vitamin D supplementation to improve disease outcomes.

## Background

Vitamin D metabolism has a well-established relationship with calcium flux and bone health [1]. Over the past decade, there has been growing interest in the non-skeletal effects of vitamin D. The anti-inflammatory and immunomodulatory effects of vitamin D have been of particular interest [2]. Vitamin D is known to be relevant in airway diseases. This includes asthma, where vitamin D supplementation in pregnancy reduced the incidence of wheezing in offspring [3, 4].

However, the role of vitamin D in chronic obstructive pulmonary disease (COPD) is less clear. COPD patients have been shown to have an increased rate of vitamin D deficiency [5], but there has not been a consistent relationship between vitamin D levels and risk of respiratory exacerbations [6]. Clinical trials [7–9] investigating the effect of vitamin D supplementation on the rate of exacerbations have yielded conflicting results. Additionally, the effects of vitamin D on imaging characteristics and clinical outcomes, including quality of life, in smokers has not been established.

This study examines the effect of vitamin D levels and vitamin D deficiency on respiratory symptoms, functional status, exacerbations, and chest CT scan characteristics in the COPDGene Study, a well-characterized study of current and former smokers with and without COPD. We hypothesized that vitamin D deficiency will be associated with worsened respiratory outcomes. We tested the association between vitamin D levels and increased exacerbations, worse quality of life, decreased lung function and exercise capacity, and CT measures of emphysema and airway disease in current and former smokers with and without COPD.

## Material and methods

### Subjects

The COPDGene study [10] is a multicenter, longitudinal observational study designed to identify subtypes and genetic factors associated with COPD. The COPDGene study includes 10,198 subjects enrolled at 21 centers in the United States. COPDGene enrolled subjects from 2008 to 2011. Study subjects were 45 to 80 years old and had a history of smoking for at least 10 pack-years at the time of enrollment. Subjects enrolled at the index visit were asked to participate in a 5-year follow up visit. Subjects who had a history of lung disease other than COPD and asthma or had had an acute respiratory exacerbation in the 30 days prior to enrollment visit were not included. Additional characteristics of study protocol, enrollment criteria, and data collection forms have been previously described and are available at www.copdgene.org.

### Data collection

Subjects completed a modified American Thoracic Society Respiratory Epidemiology Questionnaire, Modified Medical Research Council (MMRC) dyspnea scale, St. George’s Respiratory Questionnaire (SGRQ) to assess disease related quality of life, and questionnaires related to demographics and medical history. Subjects also completed a standard spirometry protocol, before and after the administration of inhaled albuterol, and a 6-min-walk test (6MWD). Chest CT scans with inspiratory and expiratory protocols were obtained. Thirona software was used for quantitative imaging analysis to determine percent emphysema (percent of voxels on inspiratory CT scan with <− 950 HU attenuation), percent gas trapping (percent of lung with <− 856 HU attenuation on expiratory CT scan), and wall thickening in segmental airways, subsegmental airways, and the square root of wall area of a hypothetical airway with 10 mm internal perimeter (Pi10) [11]. Longitudinal follow up was conducted using automated telephone contact, web-based questions, and phone call via research coordinator. Subjects enrolled in longitudinal follow up a questionnaire that asked about exacerbations, smoking status, new co-morbidities/therapies, and general health status [12]. The LIAISON 25-OH Vitamin D TOTAL Assay (DiaSorin) was used to measure plasma concentration of 25-hydroxyvitamin D (25(OH)D) in a subset of COPDGene, enriched for subjects with self-reported asthma with longitudinal data, using samples collected at the baseline study visit. The LIAISON 25-OH Vitamin D TOTAL Assay is a chemiluminescence immunoassay that is FDA approved and has been used in epidemiologic studies and clinical trials for measurement of serum 25(OH)D. Detailed assay methods have been previously published [4].

### Case definitions

COPD was defined as post-bronchodilator FEV_1_/FVC ratio < 0.70 and FEV_1_ < 80% predicted, corresponding to Global Initiative for Obstructive Lung Disease (GOLD) stages 2–4 [13]. Control subjects were defined as post-bronchodilator FEV_1_/FVC ≥0.7 and FEV_1_ ≥ 80% predicted, which has been termed GOLD 0 in previous COPDGene publications [14]. GOLD stage 1 subjects (FEV_1_/FVC < 0.7 and FEV_1_ ≥ 80% predicted were excluded from the present study. COPD exacerbations were defined by the use of systemic steroids or antibiotics. Severe exacerbations were defined by an emergency room visit or hospital admission. Vitamin D deficiency was defined as a 25(OH) D plasma concentration less than 20 ng/mL based on the Institute of Medicine recommendations [15], while subjects with 25(OH) D plasma concentrations of greater than or equal to 20 ng/mL were classified as vitamin D sufficient.

### Statistical analysis

Subjects with and without vitamin D deficiency were compared by demographics, lung function, respiratory symptoms, and CT scan measurements. Statistical analysis was performed with R (version 3.6.0). Survival analyses were performed using the survival package. Kaplan-Meier analysis was performed using the survminer R package. Multivariable regression analysis was performed, with most models adjusted for the standard covariates of age, gender, race, smoking history, FEV_1_ percent predicted, height, and body mass index; CT scanner model was also included for analyses where the outcome was a quantitative CT imaging variable. Linear regression was performed with the limma R package, and effect estimates with standard errors reported. Select analyses were also performed stratifying by race to explore the role of race as an effect modifier. Subjects with missing data were removed from specific analyses. The primary analyses were performed in all subjects to allow for case-control comparisons and to improve power for associations with quantitative outcomes. Selected secondary analyses were limited to subjects with COPD (GOLD 2–4).

## Results

### Subject characteristics

**Fig. 1:**
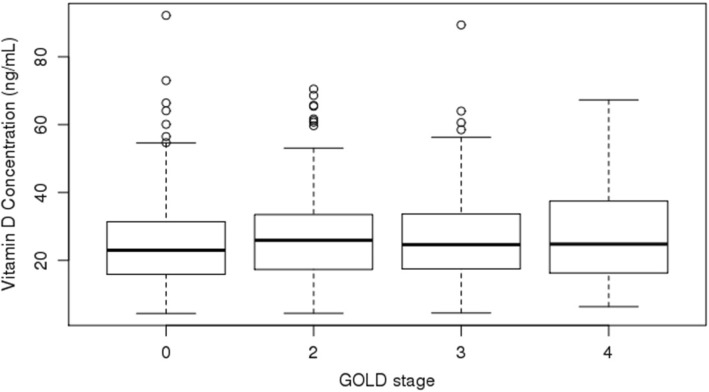
Vitamin D concentration by GOLD stage: Boxplots demonstrating the median (bold black line), middle quartiles (box), and 1st and 4th quartiles (whiskers) for vitamin D concentration, stratified by GOLD stage. There was no significant difference in median vitamin D concentration by GOLD stage

The COPDGene Study includes 10,198 current and former smokers. Serum concentrations of 25(OH) D were obtained on 1544 subjects. 981 subjects had sufficient vitamin D levels (serum 25(OH) D concentration > 20 ng/mL) and 563 subjects had vitamin D deficiency (serum 25(OH) D concentration < 20 ng/mL) (Table 1). Subjects with vitamin D deficiency were younger and more likely to be African American. There was no difference in gender distribution between the two groups. Subjects with vitamin D deficiency were also more likely to be current smokers, have higher post-bronchodilator FEV_1_ (75.9% vs 73.2% predicted), and have COPD (GOLD 2–4). There was no difference in vitamin D levels based on GOLD stage (Fig. 1).

**Table 1:** Subject Characteristics

	Vitamin D Deficient	Vitamin D Sufficient	*p* value
n	563	981	
Age in years	57.07 (7.92)	62.09 (8.67)	< 0.001
Female	302 (53.6)	520 (53.0)	0.851
Non-Hispanic White	236 (41.9)	789 (80.4)	< 0.001
African American	327 (58.1)	192 (19.6)	< 0.001
Current Smoker	352 (62.5)	385 (39.2)	< 0.001
Pack-years smoking	42.26 (25.99)	44.83 (24.56)	0.053
Post-bronchodilator FEV1 percent predicted	75.96 (25.01)	73.24 (24.86)	0.039
COPD	291 (51.8)	572 (58.3)	0.015

Mean (SD) or *N*(%) are shown

### Respiratory symptoms, functional status, and exacerbations

Vitamin D deficiency was associated with worse quality of life based on higher SGRQ total score and greater dyspnea based on higher MMRC score at baseline (Table 2). The effect on SGRQ score persisted in multivariable linear regression analysis, corresponding to a one-point decrease in SGRQ score for 5 ng/mL increase in 25(OH) D serum concentration. In addition, the effect on SGRQ score persisted in a subset of the study cohort of only subjects with COPD (Table S2). While vitamin D deficiency was not associated with reduced BODE index, vitamin D deficiency was associated with reduced exercise capacity on 6-min walk test (1313 vs 1400 ft). While the effect of vitamin D deficiency did not persist in multivariable regression, the 6MWD was increased by 2.25 ft per unit increase in serum 25(OH) D concentration (Table 3). There was no association with vitamin D levels and change in FEV_1_ over 5 years.

**Table 2:** Respiratory symptoms, functional status, and exacerbations

	Vitamin D Deficient	Vitamin D Sufficient	*p* value (Univariate analysis)	Vitamin D Deficiency Effect Estimate^b^	*p* value (Multivariable regression)
n^a^	534	957			
SGRQ total score	33.23 (23.33)	26.89 (21.19)	< 0.001	3.14 (1.12)	0.005
Chronic Bronchitis	113 (21.2)	204 (21.3)	1.0	0.91 [0.68, 1.22]	0.54
BODE score	1.68 (1.75)	1.50 (1.72)	0.068	0.061 (0.07)	0.38
MMRC dyspnea score	1.67 (1.46)	1.38 (1.42)	< 0.001	0.11 (0.074)	0.16
6 min walk distance, feet	1312.76 (366.38)	1400.16 (361.25)	< 0.001	−31.0 (18.9)	0.1
Exacerbations per year	0.43 (0.76)	0.43 (0.72)	0.94	0.032 (0.041)	0.44
Severe exacerbations per year	0.19 (0.52)	0.14 (0.32)	0.038	0.024 (0.023)	0.3

Mean (SD) or N(%) are shown

^a^Longitudinal follow up cohort had 1491 subjects, due to missing data

^b^Covariates in linear and logistic regression include age, race, gender, current smoking status, smoking pack years, and FEV_1_ percent predicted. Beta (SE) or Odds Ratio (95% CI) are shown

**Table 3:** Regression analyses showing effect of vitamin D levels on respiratory symptoms, functional status, and exacerbations^a^

	Vitamin D Effect Estimate^b^	SE or 95% CI	*p* value
COPD Diagnosis	1.02	[0.94, 1.10]	0.66
FEV_1_ percent predicted^c^	0.024	0.056	0.66
SGRQ total score	− 0.19	0.045	< 0.001
BODE score	−0.003	0.0028	0.29
MMRC score	−0.0093	0.0030	0.002
6 min walk distance, feet	2.25	0.76	0.0032
Exacerbations per year	−0.0027	0.0017	0.11
Severe Exacerbations per year	−0.0019	0.00095	0.046

^a^Each row represents the outcome of a separate regression model

^b^Covariates include age, race, gender, current smoking status, smoking pack years, and FEV_1_ percent predicted. Beta (SE) or Odds Ratio (95% CI) are shown

^c^Covariates include age, race, gender, height, current smoking status, and smoking pack years

**Fig. 2:**
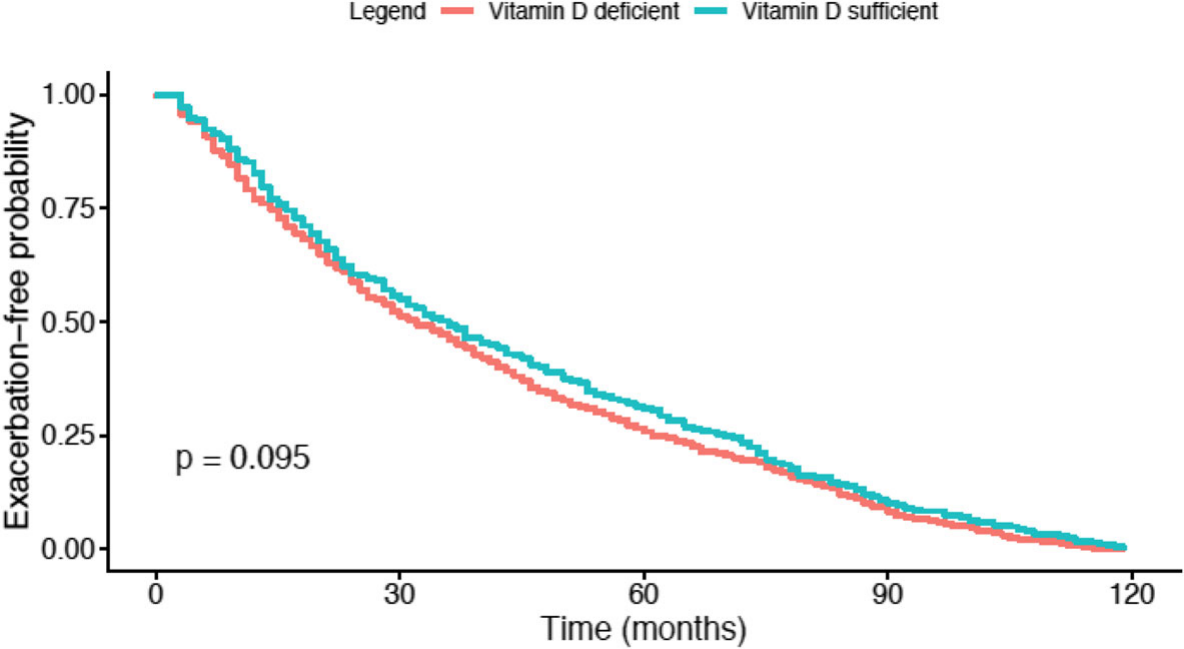
Time to exacerbation; Kaplan-Meier curves demonstrating the time to exacerbation in the vitamin D sufficient group (blue line) and the vitamin D deficient group (orange line). There was no significant difference between to two groups (*p* = 0.095)

There were 1491 subjects in the longitudinal follow up cohort (Table 2). Vitamin D deficiency was associated with more severe exacerbations per year (0.19 vs 0.14). In multivariable analysis in the longitudinal follow-up, the effect of increased 25(OH) D on reduced yearly severe exacerbation rate persisted. This finding was not recapitulated in the subset of subjects with COPD (Table S2). The median time to exacerbation was 32 months in the vitamin D sufficient group while the median time to exacerbation was 36 months in the vitamin D deficient group. However, there was no difference in time-to-event analysis of all exacerbations between the two groups (Fig. 2).

### Imaging characteristics

#### Measures of emphysema

Compared to the vitamin D deficient group, subjects with sufficient 25(OH) D serum levels had higher percent emphysema and percent gas trapping on CT at baseline (Table 4). However, these differences did not persist on multivariable analysis adjusted for age, race, gender, BMI, current smoking status, and scanner model. There was an association between higher vitamin D levels and reduced progression in gas trapping between the two visits (Table 5). On multivariable regressions stratified by race, higher vitamin D levels were associated with increased percent gas trapping in non-Hispanic white subjects with no significant effect observed in African American subjects (Table S1).

**Table 4:** Imaging characteristics^a^

	Vitamin D Deficient	Vitamin D Sufficient	*p* value (Univariate analysis)	Vitamin D Deficiency Effect Estimate (SE)^d^	*p* value (Multivariable regression)
n^b^	549	970			
% emphysema	5.80 (8.33)	7.33 (9.48)	0.002	0.76 (0.49)	0.12
15th percentile of lung density histogram (+ 1000 HU)	85.70 (32.25)	76.81 (28.45)	< 0.001	−2.1 (1.42)	0.14
% gas trapping	21.47 (18.76)	24.99 (19.20)	0.002	1.81 (1.05)	0.085
Segmental airway wall thickness	1.08 (0.23)	1.05 (0.22)	0.019	0.027 (0.012)	0.021
Pi10^c^	2.44 (0.63)	2.37 (0.59)	0.053	0.058 (0.036)	0.11
Segmental wall area %	52.03 (8.65)	51.07 (8.21)	0.040	0.81 (0.48)	0.092
Change in % emphysema	0.88 (3.87)	0.56 (4.21)	0.17	0.36 (0.25)	0.16
Change in % gas trapping	3.44 (9.89)	2.01 (9.09)	0.024	0.73 (0.64)	0.26
Change in segmental airway wall thickness	−0.00 (0.13)	0.01 (0.11)	0.12	−0.0052 (0.0077)	0.50

Mean (SD) or N(%) are shown

^a^Each row represents the outcome of a separate regression model

^b^CT scan data was available for 1519 subjects

^c^square root of wall area of hypothetical airway with 10 mm internal perimeter

^d^Covariates in linear regression models include age, race, gender, BMI, current smoking status, and scanner model

**Table 5:** Vitamin D concentration effects on imaging characteristics^a^

	Vitamin D Effect Estimate^c^	Standard Error	*p* value
% emphysema	−0.018	0.020	0.36
15th percentile of lung density histogram (+ 1000 HU)	0.11	0.059	0.074
% gas trapping	−0.012	0.043	0.78
Segmental airway wall thickness	−0.0013	0.00049	0.0085
Pi10^b^	−0.0017	0.0015	0.26
Segmental wall area %	−0.033	0.020	0.098
Change in % emphysema	−0.020	0.011	0.056
Change in % gas trapping	−0.065	0.0263	0.014
Change in segmental airway wall thickness	−0.00014	0.00032	0.65

^a^Each row represents the result of a separate regression model

^b^square root of wall area of hypothetical airway with 10 mm internal perimeter

^c^Covariates in linear regression models include age, race, gender, BMI, current smoking status, and scanner model

#### Airway measures

Vitamin D deficiency was associated higher segmental airway wall thickness (1.08 mm vs 1.05 mm) at baseline (Table 4). In regression analysis, higher vitamin D levels were associated with decreased segmental airway wall thickness. In the subset of subjects with COPD, there was no statistically significant association between vitamin D level and segmental airway wall thickness (Table S2). In analyses stratified by race, higher vitamin D levels were associated with decreased segmental airway wall thickness in both African American and non-Hispanic white subjects (Table S1). Higher vitamin D levels were also associated with lower percent segmental wall area in non-Hispanic white subjects.

## Discussion

In adult smokers with and without COPD, vitamin D deficiency was associated with increased respiratory symptoms and worse health-related quality of life at baseline, as well as increased frequency of exacerbations and airway wall thickening on chest CT scans. To our knowledge, the association of vitamin D deficiency with increased airway wall thickness on chest CT scans has not been previously reported.

The association between vitamin D and lung function has been inconsistent in the existing literature. Previous cross-sectional studies have shown a positive association with vitamin D levels and FEV_1_ [5, 16]. However, a longitudinal study of smokers with COPD showed no association in baseline vitamin D level between subjects with rapid rate of lung function decline versus those with a slow lung function decline [17]. Notably, the longitudinal cohort consisted primarily of Caucasian subjects. Another cross-sectional study of older subjects in the UK did not demonstrate an association of vitamin D level with lung function or COPD, though 46% of the study cohort were never smokers and only 17% carried a diagnosis of COPD [18]. A meta-analysis showed that vitamin D levels were inversely correlated with risk of COPD and severity of COPD [19], though the majority of the included studies consisted of subjects in China. Our data did not demonstrate an association with vitamin D levels and change in FEV_1_ over 5 years. Similarly, a large, prospective general population study found an inverse association with vitamin D status and prevalent COPD but no association between vitamin D status and incident COPD [20].

COPDGene also included measures of patient reported outcomes and functional status. There have been limited studies on the association of vitamin D levels and quality of life, respiratory symptoms, and functional status. A small randomized, placebo-controlled trial of sixty-two patients hospitalized with acute exacerbation of COPD in Iran showed significant improvement in SGRQ total score at day 30 and day 120 following hospital admission, vitamin D supplementation and normalization of vitamin D concentration [21]. Another small trial of fifty-one patients in Northern Ireland showed improved SGRQ total score with higher vitamin D levels [22]. Our data similarly showed vitamin D deficiency was associated with higher SGRQ total score, indicating worse quality of life, and higher MMRC score, consistent with increased dyspnea. In addition, our study demonstrated a positive association between vitamin D levels and six-minute walk distance.

There are many factors that contribute to the pathophysiology of acute exacerbations of COPD, including inflammatory dysregulation. Given the proposed mechanism of vitamin D as an immunomodulator, there has been interest in its role as a potentially modifiable target in the prevention of COPD exacerbations. Several retrospective and prospective cohort studies and small randomized control trials have investigated the relationship between vitamin D deficiency and acute exacerbations of COPD. A prospective cohort study showed no association with baseline vitamin D levels and exacerbation rate or time to first exacerbation [23]. A randomized, placebo-controlled trial of two hundred forty subjects with COPD showed supplementation of vitamin D reduced severe exacerbations in subjects with baseline vitamin D concentrations less than 30 ng/mL but did not affect time to first exacerbation [8]. Another randomized control trial of one hundred eighty two subjects with COPD showed no difference in time to first exacerbation or exacerbation rate between the treatment and control groups, though subgroup analysis did show a reduction in exacerbation rate with vitamin D supplementation for subjects with severe (< 10 ng/mL) vitamin D deficiency [9]. A meta-analysis, which included the studies mentioned previously, showed that vitamin D levels were inversely correlated with COPD exacerbations, but did not find an association between vitamin D deficiency and COPD exacerbations [19]. Our study similarly found that both lower vitamin D level were associated with increased frequency of severe exacerbations, but not with the frequency of all exacerbations. This result was not recapitulated in the subset of subjects with COPD, possibly due to decreased statistical power from the lower number of subjects or a low event rate. These findings could suggest that, while vitamin D may not play a role in the initiation of acute exacerbations of COPD, it may contribute to the severity of inflammation once triggered.

A unique aspect of our study was the assessment of chest CT imaging characteristics. A pilot study from the Evaluation of COPD Longitudinally to Identify Predictive Surrogate Endpoints (ECLIPSE) cohort showed an inverse association with vitamin D levels and percent emphysema on CT, though the association did not persist when limited to subjects with COPD [24]. In addition, the study subjects were predominantly (> 95%) white. Another study in the Korean Obstructive Lung Disease cohort, published as an abstract, found significant differences in CT emphysema indices between subjects with COPD stratified by severity of vitamin D deficiency [25]. We did not find a significant association between vitamin D levels and CT measures of emphysema.

Previous studies have demonstrated associations between imaging characteristics and clinical outcomes. In particular, increased segmental bronchial wall thickness and percent segmental wall area have been associated with increased risk of COPD exacerbation and impaired respiratory quality of life [26, 27]. We found that lower vitamin D levels were associated with increased segmental airway wall thickness, and the association persisted in analysis stratified by race. This finding may have implications on the role of vitamin D levels as a risk factor for COPD exacerbations as well as the contribution to clinical and imaging phenotypes.

The primary limitation of the current study is the single measurement of vitamin D in older subjects from the COPDGene Study population. The ideal study would follow younger smokers and their vitamin D levels, lung function, functional status, respiratory symptoms, and imaging over time. In addition, investigating the genetics and gene expression related to vitamin D metabolism in smokers and in subjects with COPD may help elucidate underlying mechanisms. Despite our study design, our findings are unlikely due to reverse causation. While subjects with more severe clinical disease may have lower vitamin D levels due to decreased sun exposure, the presence of airway imaging characteristics versus emphysema should not have a differential effect. Despite several clinical trials, the role of vitamin D supplementation in COPD remains unclear. Furthermore, it is unclear if the role of vitamin D rests more in the development of COPD versus the development of COPD exacerbations.

There remains a paucity of data on many aspects of the use of vitamin D. While several studies and guideline recommendations have established vitamin D sufficiency for bone health as a serum concentration greater than 20 ng/mL, the optimal vitamin D level for lung diseases has not been established. Furthermore, while 25(OH) D is the most commonly used marker for vitamin D level, it is unknown if this is the most consequential molecule in the vitamin D pathway. In addition, it is known that vitamin D levels can vary based on latitude, season, race, diet, and supplementation [1]. We were unable to control for season, diet, or supplementation in our study cohort.

## Conclusions

We found that vitamin D deficiency was associated with increased respiratory symptoms, decreased functional status, and increased frequency of severe exacerbations. This was supported by findings of increased segmental airway wall thickness on chest CT scans. Further investigations will be required to elucidate the role of vitamin D metabolism on both the development of COPD and disparate phenotypes as well as its role in development and prevention of COPD exacerbations.

## Supplementary information

**Additional file 1: Table S1**. Regression analyses on selected outcomes stratified by race.

**Additional file 2: Table S2.** Effect of vitamin D levels on select outcomes in COPD cases only.

## Data Availability

COPDGene data are available in the NCBI Database of Genotypes and Phenotypes (dbGaP), accession phs000179.v6.p2. [https://www.ncbi.nlm.nih.gov/projects/gap/cgi-bin/study.cgi?study_id=phs000179.v6.p2].

## Abbreviations

COPD: Chronic obstructive pulmonary disease
COPDGene: Genetic epidemiology of COPD study
25(OH) D: 25-hydroxyvitamin D
CT: Computed tomography
FEV_1_: Forced expiratory volume in 1 s
MMRC: Modified Medical Research Council dyspnea scale
SGRQ: St. George’s Respiratory Questionnaire
6MWD: 6-min-walk test
HU: Hounsfield unit
Pi10: Square root of wall area of a hypothetical airway with 10 mm internal perimeter
FDA: Food and Drug Administration
FVC: Forced vital capacity
GOLD: Global Initiative for Chronic Obstructive Lung Disease
BODE: Body-Mass Index, Airflow Obstruction, Dyspnea, and Exercise Capacity Index
BMI: Body-Mass Index
UK: United Kingdom
ECLIPSE: Evaluation of COPD Longitudinally to Identify Predictive Surrogate Endpoints Study
