# Supplementary material for: Vitamin D deficiency is associated with respiratory symptoms and airway wall thickening in smokers with and without COPD: a prospective cohort study
Source: BMC Pulm Med. 2020 May 4;20:123. doi: 10.1186/s12890-020-1148-4 (PMC7199369; doi:10.1186/s12890-020-1148-4)
Supplement: Supplementary file 1 — Additional file 1: Table S1. Regression analyses on selected outcomes stratified by race. [file 12890_2020_1148_MOESM1_ESM.docx]

| **Table S1: Regression analyses on selected outcomes stratified by race*** | | | |  |
| --- | --- | --- | --- | --- |
| *Response Variable* | *Vitamin D Estimate, African American*** | *p value* | *Vitamin D Estimate, Non-Hispanic White*** | *p value* |
| % emphysema | 0.044 | 0.14 | 0.033 | 0.14 |
| 15th percentile of lung density histogram (+1000 HU) | 0.025 | 0.85 | -0.13 | 0.048 |
| % gas trapping | 0.040 | 0.55 | 0.13 | <0.001 |
| Segmental airway wall thickness | -0.0019 | 0.063 | -0.0018 | <0.001 |
| Pi10 | 0.00037 | 0.88 | -0.0018 | 0.16 |
| Segmental wall area % | -0.013 | 0.72 | -0.060 | 0.0019 |
| Exacerbations per year | -0.0018 | 0.6 | -0.0029 | 0.14 |
| Severe Exacerbations per year | -0.0034 | 0.18 | -0.0014 | 0.11 |
| SGRQ total score | -0.16 | 0.13 | -0.20 | <0.001 |
| BODE score | 0.0068 | 0.26 | -0.0066 | 0.039 |
| MMRC score | 0.0016 | 0.81 | -0.013 | <0.001 |
| 6 min walk distance, feet | 0.24 | 0.88 | 3.21 | <0.001 |
