# Supplementary material for: Vitamin D deficiency is associated with respiratory symptoms and airway wall thickening in smokers with and without COPD: a prospective cohort study
Source: BMC Pulm Med. 2020 May 4;20:123. doi: 10.1186/s12890-020-1148-4 (PMC7199369; doi:10.1186/s12890-020-1148-4)
Supplement: Supplementary file 2 — Additional file 2: Table S2. Effect of vitamin D levels on select outcomes in COPD cases only. [file 12890_2020_1148_MOESM2_ESM.docx]

| **Table S2: Effect of vitamin D levels on select outcomes in COPD cases only*** | | | |
| --- | --- | --- | --- |
|  | *Vitamin D Effect Estimate* | *Standard Error* | *p value* |
| SGRQ total score** | -0.17 | 0.06 | 0.0047 |
| Severe Exacerbations per year** | -0.02 | 0.01 | 0.075 |
| Segmental airway wall thickness*** | -0.0009 | 0.0006 | 0.17 |
